# SARS-CoV-2 Ultraviolet Radiation Dose-Response Behavior

**DOI:** 10.6028/jres.126.018

**Published:** 2021-08-20

**Authors:** Ernest R. Blatchley III, Brian Petri, Wenjun Sun

**Affiliations:** 1 Lyles School of Civil Engineering, Purdue University, West Lafayette, IN 47907, USA; 2 Environmental and Ecological Engineering, Purdue University, West Lafayette, IN 47907, USA; 3 Trojan Technologies, London, Ontario N5V 4T7, Canada; 4 School of Environment, Tsinghua University, Beijing 100084, China

**Keywords:** COVID-19, disinfection, SARS-CoV-2, ultraviolet radiation, virus

## Abstract

Ultraviolet (UV) radiation in the wavelength range 200 nm ≤ λ ≤ 320 nm, which includes both the UV-C and UV-B portions of the spectrum, is known to be effective for inactivation of a wide range of microbial pathogens, including viruses. Previous research has indicated UV-C radiation to be effective for inactivation of severe acute respiratory syndrome coronavirus (SARS-CoV), the virus that caused an outbreak of SARS in 2003. Given the structural similarities of SARS-CoV and SARS-CoV-2, the cause of coronavirus disease 2019 (COVID-19), it is anticipated that UV radiation should be effective for inactivation of SARS-CoV-2 too. Recently published data support this assertion, but only for a narrow set of exposure and matrix conditions. Models based on genomic and other characteristics of viruses have been developed to provide predictions of viral inactivation responses to UV exposure at λ = 254 nm. The predictions of these models are consistent with reported measurements of viral inactivation, including for SARS-CoV-2. As such, current information indicates that UV-C irradiation should be effective for control of SARS-CoV-2, as well as for control of other coronaviruses; however, additional research is needed to quantify the effects of several important process variables, including the wavelength of radiation, the effects of relative humidity on airborne and surface-associated viruses, and the effects of the medium of exposure.

## 1 Introduction

Ultraviolet (UV) radiation is a broad-spectrum antimicrobial/antiviral agent that has been applied successfully in a wide range of disinfection applications. UV radiation in the wavelength range 200 nm ≤ λ ≤ 320 nm, sometimes referred to as “germicidal” or “microbicidal” UV radiation, is known to cause damage to DNA and RNA that results in inactivation of microorganisms and viruses. For radiation with wavelengths less than about 240 nm, damage to proteins can also contribute to inactivation [1–3]. Given that all viruses contain a nucleic acid molecule, either DNA or RNA, and a protein coat (capsid) that surrounds the nucleic acid, all viruses are susceptible to inactivation by exposure to UV-C radiation. However, viral sensitivity to UV-C radiation is quite variable; the development of a comprehensive understanding of the causative factors is still an active area of research.

Severe acute respiratory syndrome coronavirus 2 (SARS-CoV-2; also known as the novel coronavirus) is the virus that causes coronavirus disease 2019 (COVID-19). Transmission of COVID-19 appears to be largely associated with airborne particles that may be released by symptomatic or asymptomatic individuals who have been infected by SARS-CoV-2, although it is also known that the virus can remain infective on surfaces for as much as 24−72 h, depending on the material it is contact with [4], so contact with contaminated surfaces (fomites) represents another possible mechanism of disease transmission [5].

At present, only limited data are available to define how SARS-CoV-2 responds to common disinfectants, including UV radiation. UV dose-response behavior describes the intrinsic kinetics of UV inactivation, and as such, it represents a key piece of information for the design of UV disinfection systems that are intended for inactivation of SARS-CoV-2. The goal of this paper is to present a summary of information that was available at the time of publication to describe inactivation of SARS-CoV-2 by exposure to germicidal UV radiation.

## 2 Dose-Response Data for Coronaviruses

Most UV dose-response experiments have involved exposure of the target microbe or virus to UV radiation while suspended in an aqueous medium. These experiments tend to be relatively easy to conduct and analyze. Moreover, the data from these experiments can be used to inform the design and analysis of UV disinfection systems that are used in treatment of water, which historically have been the most common applications of UV disinfection processes.

The simplest and most commonly applied model to describe UV dose-response behavior (i.e., UV disinfection kinetics) of microbes and viruses is the single-event model, which implies that a single unit of photochemical damage is sufficient to inactivate a microbial or viral target. The single-event model, which implies first-order kinetics, takes the following mathematical form:

$\frac{dN}{dt}=-kEN$

where *N* = concentration of viable or infective microbe or virus, *t* = time, *k* = 1^st^-order inactivation constant, and *E* = fluence rate. Separation of variables and integration yields a common form:

$ln\left(\frac{N}{N_{0}}\right)=-kD$

where *N_0_* = concentration of viable or infective microbes or viruses before exposure to UV radiation and *D* = UV dose, which may also be represented as the product of fluence rate and exposure time. Dose is often expressed in units of mJ/cm^2^, while the inactivation constant will have units that are the inverse of those used to quantify dose, cm^2^/mJ.

Until recently, no data were available to describe the responses of SARS-CoV-2 to germicidal UV radiation. However, several studies that were performed prior to the start of the COVID-19 pandemic reported UV dose-response behavior of SARS-CoV; this is the virus that caused an epidemic of severe acute respiratory syndrome (SARS) that affected roughly 8000 people in 26 countries in 2003 [6]. SARS-CoV is closely related to SARS-CoV-2, with both viruses belonging to the coronavirus family. SARS-CoV and SARS-CoV-2 are both enveloped, single-stranded (ss), positive-sense RNA viruses, and they share roughly 80% similarity in terms of their genomes [7, 8].

SARS-CoV-2 belongs to the *Coronaviridae* family, which includes the largest known ssRNA viruses [9]. Coronaviruses (CoV) range in size from 118 to 140 nm, with genome size of 25−32 kilobases (kb). Seven coronaviruses are known to cause disease in humans. These include four viruses that cause the “common cold” (HCoV-229E, HCoV-NL63, HCoV-OC43, and HCoV-HKU1) [10, 11]. Three coronaviruses have been identified that cause more serious, sometimes fatal diseases in humans: SARS-CoV, MERS-CoV (cause of Middle East respiratory syndrome, MERS), and SARS-CoV-2. The structural similarity of these viruses, including their relatively large genomes, suggests that they should all be susceptible to inactivation by exposure to UV-C radiation and that their UV-C dose-response behaviors should be similar [10–15].

Studies of the responses of SARS-CoV published to date have been based on UV-C radiation at or near the wavelength 254 nm (UV_254_), which characterizes the output of low-pressure mercury lamps, which are the most commonly used source of germicidal UV radiation [16–18]. A wide range of responses was reported among the studies, and all had deficiencies in their experimental methods. The reported fluences (doses) were probably overestimates, as UV absorbance of the suspensions was not reported or explicitly accounted for in the experiments. Similarly, the methods used to irradiate the viral suspensions suggest that the UV dose applied could not be accurately calculated. Specifically, in each of these studies, radiation was delivered from a UV-C source in a manner that did not allow for accurate measurement of the applied fluence rate by conventional methods, such as radiometry. Although the results of these studies do not appear to provide accurate information regarding the UV_254_ dose-response behavior of SARS-CoV, all three studies reported measurable inactivation of the virus to result from UV_254_ irradiation.

Gerchman *et al*. [19] conducted a set of experiments to define the dose-response behavior of HCoV-OC43 to radiation from various UV light-emitting diodes (LEDs). Specifically, they used UV LEDs with peak output at nominally 267 nm, 279 nm, 286 nm, and 297 nm, with full-width at half-maximum (FWHM) bandwidths of roughly 12−20 nm. Their experimental design, which involved methods of exposure and dose calculation that were new and somewhat unconventional, resulted in a limit of quantification of 3 log_10_ units of inactivation of the target virus.^1^1 3 log_10_ units refers to a 99.9% reduction, calculated as log_10_ (*N*_0_/*N*), where *N*_0_ is the initial value, and *N* is the final value. Regression of their data that were within the limit of quantification using a single-hit (first-order) model of disinfection kinetics allowed estimation of inactivation constants for HCoV-OC43 as a function of wavelength. These estimates of wavelength-dependent inactivation behavior are summarized in Table 1. Because the genome for HCoV-OC43 is similar in size to that of SARS-CoV-2, and because they are both betacoronaviruses in the *Coronaviridae* family, it is anticipated that their responses to UV-C radiation will be similar. As such, HCoV-OC43 appears to represent a good surrogate for SARS-CoV-2.

**Table 1:** Estimates of first-order UV inactivation constants for HCoV-OC43 from data reported by Gerchman *et al*. [19]. Estimates of inactivation constants were developed by regression of data that were within the limit of quantification using a single-hit (first-order) model.

Wavelength (nm)	Inactivation Constant (cm^2^/mJ)
267	0.77
279	0.64
286	0.43
297	0.14

**Fig. 1:**
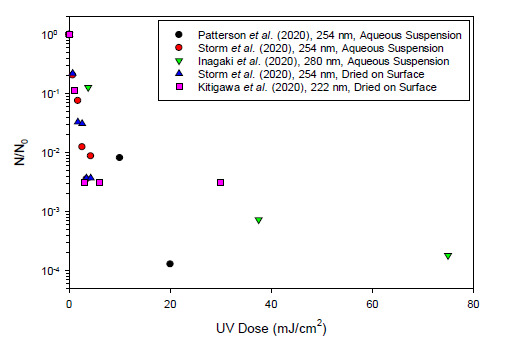
Reported UV dose-response data for SARS-CoV-2 in aqueous suspension and dried on surfaces [21–24]. *N* is the final value; *N*_0_ is the initial value; 3 log_10_ units of inactivation is represented by 10^−3^. Note that results are reported for several different wavelengths of UV-C radiation. The nominal wavelength of imposed radiation and the conditions of virus exposure to UV-C radiation are indicated in the legend. The data reported for 254 nm are all from experiments that involved the use of low-pressure Hg lamps as the source of radiation, with effectively monochromatic output at 254 nm. The data reported for 280 nm are based on exposure to radiation from a UV LED with peak output at that wavelength. The data reported for 222 nm are based on exposure to radiation from a KrCl* lamp with an optical filter used to eliminate all radiation except the dominant peak near 222 nm.

Since the start of the COVID-19 pandemic, several research groups have undertaken efforts to quantify the UV-C dose-response behavior of SARS-CoV-2. To date, the results of these efforts have appeared in a wide range of publications, including commercial advertisements, press releases, prepublications, and the refereed literature. Figure 1 provides a summary of data from peer-reviewed papers in which it was possible to define, in quantitative terms, the inactivation response of SARS-CoV-2 as a function of applied UV-C dose. However, even in these papers, there remains some ambiguity as to how UV radiation was delivered to the viral targets and how the reported doses were calculated. As with the SARS-CoV work, there was a wide range of responses; data from studies that were judged to indicate likely false-high resistance [20] were excluded from Fig. 1.

Note that in Fig. 1, three of the data sets indicate inactivation responses for the virus suspended in an aqueous medium, while the other two data sets indicate responses of the virus after being applied to a surface as an aqueous suspension and then allowed time to air dry before UV-C exposure. Note also that three of the investigations were conducted using low-pressure Hg lamps as the source of radiation (λ = 254 nm), while one investigation was based on UV LED (peak λ = 280 nm), and another was based on a Krypton Chloride excimer (KrCl*) lamp as the source of radiation (peak λ = 222 nm). Collectively, the data presented in these recent papers indicate that SARS-CoV-2 is quite sensitive to germicidal UV radiation, which is consistent with the behavior of related viruses and the known structure of SARS-CoV-2.

## 3 Action Spectra

The effectiveness of UV-C radiation as a disinfectant is influenced by the wavelength of radiation. This behavior has become increasingly important in recent years with the development of alternatives to conventional low-pressure mercury lamps, including medium-pressure mercury lamps, UV LEDs, and plasma (excimer) lamps, all of which are polychromatic and have output that varies substantially from 254 nm. The wavelengths of radiation produced by LEDs and excimers depend on their chemical composition. Collectively, these alternative sources provide access to radiation from across the germicidal UV spectrum.

**Fig. 2:**
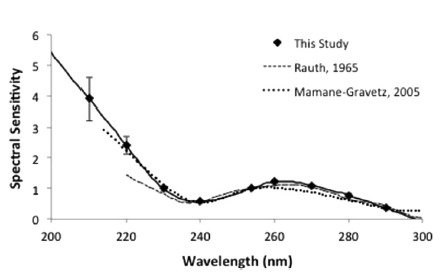
Normalized action spectrum for coliphage MS2 (figure from Beck *et al*. [25], indicated as “This Study”; figure reprinted with permission). For all three data sets presented in this graph, the data were normalized against the measured response at 254 nm. Also included in this figure are action spectra from Rauth [26] and Mamane-Gravetz [27]. Error bars represent one standard deviation from the mean sensitivity value; *n* = 4 for 240 nm, 253.7 nm, 260 nm, and 270 nm, and *n* = 3 for all other wavelengths tested.

A common graphical method for describing the effects of wavelength on microbial inactivation is the so-called “action spectrum.” In most cases, the action spectrum illustrates the relative rate of inactivation of a microbe at a given wavelength compared to its inactivation rate in response to irradiation at 254 nm. An example of a normalized action spectrum for coliphage MS2 in aqueous suspension is presented in Fig. 2. As with most action spectra, the information presented in Fig. 2 indicates that for wavelengths in the range 240 nm to 300 nm, peak inactivation efficiency is obtained at about 265 nm, with steady decreases at wavelengths above and below this peak. In this wavelength range, the majority of viral inactivation is attributable to photochemical damage to its nucleic acid. For radiation at wavelengths less than about 240 nm, a rapid increase in the efficiency of inactivation occurs; this is attributable to damage to proteins, which is known to take place at these short wavelengths. Damage to nucleic acids also takes place at these relatively short wavelengths, so viral inactivation in this range is attributable to the combined effects of damage to nucleic acids and proteins. It is expected that SARS-CoV-2 will display similar trends, but insufficient data are available at present to confirm or refute this hypothesis.

The ultimate disinfection efficacy in an actual application will also be influenced by the absorbance characteristics of the medium that is being disinfected. In some settings, there could be substances that absorb strongly at wavelengths below 240 nm, which will mitigate the contributions of short-wavelength UV-C radiation.

At present, no information is available to define the action spectrum of SARS-CoV-2. Development of an action spectrum for this virus will represent an important contribution to the effort to control the virus, especially in indoor settings. This information is needed to provide a quantitative description of the response of SARS-CoV-2 to radiation from the wide range of UV-C sources that are available today. The data from the work of Gerchman *et al*. [19] will be useful in evaluating the action spectrum of coronaviruses in general and should serve as a guide for future experiments designed to develop an action spectrum for SARS-CoV-2, at least for wavelengths above 267 nm.

## 4 Effects of the Medium

**Fig. 3:**
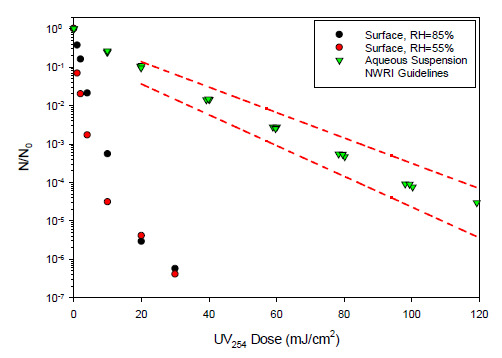
UV_254_ dose-response behavior of coliphage MS2 on gel surfaces (at two different values of RH) and in aqueous suspension. Data for MS2 on surfaces are from Tseng and Li [32]. Data for MS2 in aqueous suspension were provided by HDR/HydroQual (O. Karl Scheible and Chengyue Shen, personal communication). Figure also shows guideline limits for MS2 UV_254_ dose-response behavior, as suggested by the National Water Research Institute (NWRI) [41].

Most UV dose-response data have been reported for experiments wherein the target virus was suspended in water. These experiments are critical for UV disinfection of water; however, the transmission of SARS-CoV-2 (and many other viruses) generally involves aerosolized viruses that are suspended in air or attached to surfaces. For both conditions, the virus may experience drying (desiccation). Desiccation, which will result from exposure to air and will be influenced by relative humidity (RH), is known to represent a form of stress for most microbes and viruses, and it may alter their sensitivity to other forms of environmental stress, including UV-C exposure, as illustrated in Fig. 3 for coliphage MS2 [28–32]. Similar trends have been reported for other aerosolized viruses, including vaccinia virus and influenza H1N1 virus [33, 34]. However, it should be noted that the effects of RH on coronaviruses and other airborne or surface-associated viral pathogens remain somewhat unclear, in that some studies have indicated that these viruses survive longer at low RH [35–37], while others indicate that they survive longer at high RH [38], and still others indicate a non-monotonic association between virus survival and RH [39] or no correlation at all [40]. As such, the effects of RH on airborne viruses and viruses on surfaces, including SARS-CoV-2, represent a subject for continued research.

Other features included in Fig. 3 are the limits for MS2 inactivation suggested by the National Water Research Institute [41]. These limits provide an indication of the variability that can be expected for reported values of viral (or microbial) UV-C dose-response behavior, even for experiments that conform to all relevant experimental protocols. As such, it may be reasonable to expect similar variability to emerge in SARS-CoV-2 UV-C dose-response data.

## 5 Exposure to Solar UV-B Radiation

Solar UV-B radiation is known to function as an effective disinfectant for inactivation of a wide range of microorganisms, including bacteria, viruses, and protozoa [42–44]. Inactivation of microbial and viral pathogens by exposure to solar UV-B radiation represents an important contributing factor in solar UV disinfection processes that are often used for production of drinking water in developing countries [45, 46].

A study conducted by scientists at the U.S. Department of Homeland Security indicated that UV-B radiation in ambient sunlight plays an important role in the environmental fate and stability of SARS-CoV-2 [47]. Specifically, for solar irradiation at 40°N latitude, their measurements and modeling results indicated that 1 log_10_ unit of inactivation would be achieved for SARS-CoV-2 suspended in saliva, after allowing for drying on the surface, when exposed to 6.8 min of midday sunlight on the summer solstice. By contrast, the same extent of inactivation would require 14.3 min of exposure at the winter solstice. For perspective, the 40th parallel (north) passes close to the U.S. cities of Philadelphia, PA, Columbus, OH, Indianapolis, IN, Boulder, CO, and close to the California-Oregon border. Locations north of this line can expect to obtain slower inactivation by solar UV-B exposure, while those lying closer to the equator can expect to achieve more rapid inactivation by this mechanism.

Most commercial UV disinfection systems are developed around sources of UV-C radiation, rather than UV-B or UV-A radiation. This is largely because UV-C radiation is much more effective for inactivation of pathogens than either UV-B or UV-A radiation. Also, UV-C sources are inexpensive and relatively efficient at converting input electrical power into output UV-C radiation.

## 6 Model Predictions of SARS-CoV-2 Sensitivity to UV_254_ Exposure

Models have been developed to predict the sensitivity of viruses to UV irradiation, with particular emphasis given to solar UV-B exposure and UV_254_ radiation. Among the earliest of these efforts was the work of Lytle and Sagripanti, who developed a model to allow simulation of the sensitivity of a number of viruses that are pathogenic to humans and are of concern with respect to biodefense applications [12, 48, 49]. Their model was based on the hypothesis that within a group of viruses, for which the structure of their genome is likely to be similar, the sensitivity of a virus to UV exposure is directly proportional to genome size. The model was applied for estimation of the sensitivity of viruses to solar UV-B exposure, as well as exposure to UV-C radiation at λ = 254 nm. Their model provided estimates of viral inactivation responses that were judged by the authors to be acceptably close to measured values that had been reported in the literature. Using that approach, they were able to extend their model to estimate the sensitivity of SARS-CoV-2 to UV_254_ exposure [13]. Their model predicted an inactivation rate constant for SARS-CoV-2 of 3.3 cm^2^/mJ, based on an assumption of single-event (*i.e.*, first-order) inactivation kinetics.

Pendyala *et al*. [14] developed the *Pyrimidine Dinucleotide Frequency Value* (PyNNFV) model based on the frequency of various dinucleotide sequences within the viral genome. Their model was shown to provide accurate estimates of viral sensitivity to UV_254_ exposure over a wide range of virus types. The PyNNFV model yielded an estimate of the rate constant for SARS-CoV-2 inactivation of 1.07 cm^2^/mJ at 254 nm.

Rockey *et al*. [15] conducted a review of the literature related to UV_254_ dose-response behavior of viruses. For positive-sense, single-stranded RNA viruses, which include the coronaviruses, they developed a multiple linear regression model that yielded the lowest root-mean-squared relative prediction error (RMSrPE) based on the following variables: number of cytosines, uracils, uracil doublets, and uracil triplets. Their final model demonstrated RMSrPE that was lower than the error associated with measured values of inactivation constants from experiments. Their model indicated a UV_254_ inactivation constant of (2.0 ± 0.86) cm^2^/mJ for SARS-CoV-2, and similar values for other coronaviruses that have been linked to serious human diseases, including SARS-CoV and MERS-CoV.

**Fig. 4:**
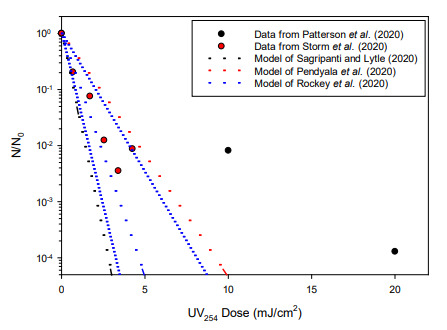
Reported UV dose-response data for SARS-CoV-2 in aqueous suspension [21, 22] by exposure to UV_254_ radiation and model predictions of SARS-CoV-2 inactivation by UV_254_ irradiation [13–15]. Dashed lines indicate mean first-order inactivation responses predicted by the models; dotted lines indicate the 95% margin of error for the model of Rockey *et al*. [15].

Figure 4 illustrates the predictions of these three models, along with measured inactivation responses of SARS-CoV-2 at 254 nm in aqueous suspension. The models differ somewhat in their predictions of SARS-CoV-2 inactivation, but they all indicate that the virus is quite sensitive to UV_254_ irradiation. For perspective, the UV_254_ inactivation kinetics for SARS-CoV-2 inactivation predicted by these models were also similar to those that have been reported for many vegetative bacterial cells [50], which are generally considered to be easy to inactivate by UV-C irradiation. Coincidentally, the range of inactivation responses predicted by the three models is similar to the range of measured responses provided by the two reports of experimental SARS-CoV-2 inactivation by UV_254_ irradiation for aqueous suspensions of the virus [21, 22].

## 7 Summary

Available information indicates promise for the use of UV-C to inactivate SARS-CoV-2; however, it should be noted that some important details of the experiments reported to date have not been presented in the papers that have been published to describe SARS-CoV-2 inactivation by UV-C irradiation. Specifically, it is important that experimental methods be implemented in a manner that allows absorption of incident UV radiation by the surrounding medium to be accounted for, explicitly. Likewise, it is important that radiation from the UV source be applied in a manner that allows accurate quantification of the applied fluence rate and dose [51]. It is likely that publications in this area will continue to emerge, and our collective understanding of the effectiveness of germicidal UV radiation for control of SARS-CoV-2 will grow. The effects of the medium of exposure, relative humidity, and wavelength(s) of exposure also need to be quantified.

Despite these shortcomings, available evidence suggests that UV-C radiation should be effective for inactivation of SARS-CoV-2. UV-C-based systems will have important roles in battling SARS-CoV-2 in air, on surfaces, and in other media. However, like all common disinfectants (*e.g.*, UV, chlorine, ozone, hydrogen peroxide), a need exists to more clearly quantify the kinetics of inactivation for SARS-CoV-2 for these applications.
